# Leaf Saponins of *Quillaja brasiliensis* as Powerful Vaccine Adjuvants

**DOI:** 10.3390/pharmaceutics17080966

**Published:** 2025-07-25

**Authors:** Víctor Morais, Norma Suarez, Samuel Cibulski, Fernando Silveira

**Affiliations:** 1 Unidad Académica de Desarrollo Biotecnológico, Instituto de Higiene, Facultad de Medicina, Universidad de la República (Udelar), Av. Alfredo Navarro 3051, Montevideo 11600, Uruguay; vmorais@higiene.edu.uy (V.M.); nsuarezster@gmail.com (N.S.); 2 FACISA—Faculdade de Ciências da Saúde do Trairi, Universidade Federal do Rio Grande do Norte (UFRN), Av. Rio Branco, S/N, Santa Cruz 59200-000, Brazil; cibulski@cbiotec.ufpb.br

**Keywords:** *Quillaja*, saponins, vaccine adjuvants, nanoadjuvants, ISCOM-matrices

## Abstract

Vaccine adjuvants are non-immunogenic agents that enhance or modulate immune responses to co-administered antigens and are essential to modern vaccines. Despite their importance, few are approved for human use. The rise of new pathogens and limited efficacy of some existing vaccines underscore the need for more advanced and effective formulations, particularly for vulnerable populations. Aluminum-based adjuvants are commonly used in vaccines and effectively promote humoral immunity. However, they mainly induce a Th2-biased response, making them suboptimal for diseases requiring cell-mediated immunity. In contrast, saponin-based adjuvants from the *Quillajaceae* family elicit a more balanced Th1/Th2 response and generate antigen-specific cytotoxic T cells (CTL). Due to ecological damage and limited availability caused by overharvesting *Quillaja saponaria* Molina barks, efforts have intensified to identify alternative plant-derived saponins with enhanced efficacy and lower toxicity. *Quillaja brasiliensis* (A.St.-Hil. and Tul.) Mart. (syn. *Quillaja lancifolia* D.Don), a related species native to South America, is considered a promising renewable source of *Quillajaceae* saponins. In this review, we highlight recent advances in vaccine adjuvant research, with a particular focus on saponins extracted from *Q. brasiliensis* leaves as a sustainable alternative to *Q. saponaria* saponins. These saponin fractions are structurally and functionally comparable, exhibiting similar adjuvant activity when they were formulated with different viral antigens. An alternative application involves formulating saponins into nanoparticles known as ISCOMs (immune-stimulating complexes) or ISCOM-matrices. These formulations significantly reduce hemolytic activity while preserving strong immunoadjuvant properties. Therefore, research advances using saponin-based adjuvants (SBA) derived from *Q. brasiliensis* and their incorporation into new vaccine platforms may represent a viable and sustainable solution for the development of more less reactogenic, safer, and effective vaccines, especially for diseases that require a robust cellular immunity.

## 1. Introduction

Vaccines developed in the two centuries since Jenner’s time have led to remarkable reductions in infection and disease wherever implemented [1]. Today, vaccination is regarded as one of the most effective medical interventions and plays a key role in reducing the burden of infectious diseases [2,3,4]. Effective vaccines and proper immunization schedules prevent millions of deaths worldwide each year. Notably, global vaccination efforts led to the eradication of smallpox, with no cases reported since 1978 [5]. Moreover, sustained and widespread vaccination efforts have led to the eradication of certain infectious diseases, such as poliomyelitis in the Americas and many other regions globally [6]. In the last pandemic of SARS-CoV-2, vaccines proved once again to be a powerful tool to control infection, and they contributed to the reduction of serious cases that lead, in many cases, to death [7,8,9]. Unfortunately, many vaccine antigens are poorly immunogenic, requiring adjuvants to elicit protective immune responses that correlates with the enhancement of the vaccine efficacy [10]. The addition of adjuvants (from Latin adjuvare, meaning “to help”), or “helper substances”, to vaccine formulations has significantly enhanced their effectiveness and the level of protection they provide. Immunological adjuvants were first described by Gaston Ramon [11] as “substances used in combination with a specific antigen that produce more immunity than the antigen alone”. In this sense, Ramon recognized that a variety of substances could increase antigen-specific antibody production when added to diphtheria and tetanus toxoids prior to vaccination [11].

Globally, vaccine adjuvants enhance efficacy by boosting, modulating, and extending the immune response [12,13]. They can also lower the required antigen dose, improving vaccine cost-effectiveness [14,15]. Adjuvants are now considered essential to modern vaccines, yet only a few are approved for human use despite advances in antigen isolation and production. These include aluminum salts, virus-like particles (VLP), MF59™, AS01™, AS03™, AS04™, and CpG [9,12].

Aluminum salts, developed almost a century ago, remain the most commonly used adjuvant for licensed human vaccines; they are found in toxoid vaccines like the tetanus vaccine and inactivated vaccines like the Salk polio vaccine and the Coronavac (SARS-CoV-2) vaccine, among others [8,16,17,18]. Due to the limitations of alum adjuvants, other compounds have been used in some vaccines. MF59™ was the second adjuvant introduced for human use [19,20,21,22]. MF59™ is an oil-in-water squalene emulsion used in flu vaccines across Europe [23]. Its adjuvant effect is linked to the early activation of innate immunity at the injection site [19,24]. While such emulsions promote a balanced immune response, they remain weak in inducing T-helper 1 (Th1) immunity and fail to effectively stimulate CTL [21]. The Adjuvant System (AS) is a proprietary formulation by GlaxoSmithKline (GSK) that combines known adjuvant compounds. AS01™, a liposome-based system containing QS-21 (a highly purified saponin from *Q. saponaria*) and 3-O-desacyl-4′-monophosphoryl lipid A (MPL), a potent adjuvant that has been used in two licensed vaccines, Shingrix™ for herpes zoster [25] and Mosquirix™ for malaria [26,27,28,29]. AS03™ is an oil-in-water emulsion included in the influenza vaccine [30], and finally, AS04™ is an MPL-absorbed aluminum salt used for hepatitis and papilloma virus vaccines [6,31]. AS incorporates immunostimulatory substances that tailor immune responses to specific pathogens, enhancing Th1 or CTL responses when needed. Current formulations combine molecules like MPL, QS-21, and CpG with classical adjuvants such as aluminum, liposomes, or oil-in-water emulsions [12,18,29,30,32,33,34,35].

QS-21 promotes Th1 cytokines (IL-2, IFN-γ), stimulates CTL responses to exogenous antigens, and induces IgG1, IgG2b, and IgG2a antibodies. These properties have made it a promising adjuvant for vaccines targeting intracellular pathogens and therapeutic cancer vaccines [34,36,37]; CTL responses are crucial for protection against intracellular pathogens like tuberculosis, malaria, and HIV. Developing effective vaccines requires inducing strong T cell immunity, a limitation of current approaches [3,38,39]. Adjuvants may be needed to enhance both antibody and effector T cell responses [40].

Despite decades of research, the immunological mechanisms of many adjuvants remain poorly understood [18,41]. It is established, however, that adjuvants primarily activate innate immune cells [12,13], often by prolonging antigen exposure to dendritic cells (DC) and promoting their maturation [42]. The discovery of Toll-like receptors (TLR) in the 1990s helped clarify the mechanisms of certain immunostimulants, such as flagellin, MPL, and CpG [43]. Despite advances, significant knowledge gaps remain for adjuvants that do not act via known receptors, such as mineral salts, microparticles, lipid-based carriers, SBA, and immunostimulant complexes (ISCOMs) [18,44,45,46,47,48]. Alum remains the most widely used adjuvant and is effective in promoting humoral immunity [49,50,51]. However, it primarily induces Th2-type responses in mice and is suboptimal for diseases requiring strong cell-mediated immunity [16,18,50,52,53,54].

Early adjuvants were designed mainly to boost antibody responses, which was adequate for many vaccines at the time. However, it is now clear that antibody enhancement alone is insufficient for many modern vaccine candidates [55]. Adjuvant selection should be guided by the specific immune response needed, such as Th1, Th2, antibody, or CTL-driven immunity [13,18]. As a result, there is an increasing demand for safe, non-toxic adjuvants capable of eliciting strong, durable protective immune responses [12,56,57].

## 2. Saponins

### 2.1. Characteristics, Structure, and Properties

Saponins are one of the most numerous and diverse plant secondary metabolites. Saponins are widely distributed in the plant Kingdom, operating as a chemical barrier or shield in the plant defense system to counter pathogens, parasites, and herbivores [58,59]. Therefore, it is found in plant tissues that are most vulnerable to fungal or bacterial attack or insect predation [60].

**Figure 1:**
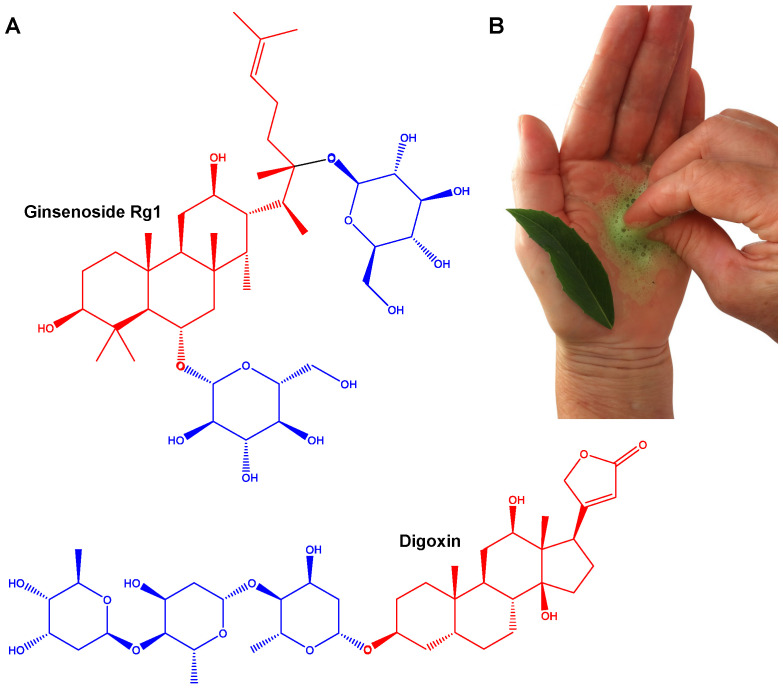
Chemical structures of representative triterpenoid and steroidal saponins. (**A**) The structural diversity of saponins, highlighting two main classes, triterpenoid saponins (e.g., ginsenoside Rg1) and steroidal saponins (e.g., digoxin). In each molecule, the aglycone (hydrophobic) is shown in red, while the sugar moieties (hydrophilic) are shown in blue. Digoxin is a cardiac glycoside derived from *Digitalis lanata* Ehrh. that is clinically used to treat heart failure and atrial fibrillation. Ginsenoside Rb1, isolated from *Panax ginseng* C.A.Mey., is a triterpenoid saponin commonly found in herbal formulations with reported immunomodulatory, neuroprotective, and anti-inflammatory effects. (**B**) Foam formed after crushing the leaves of *Q. brasiliensis* (“pau-de-sabão”, which means soap tree) in water (Photo: Iracema Rubas Cibulski).

The name saponin is derived from the Latin word sapo, which means soap, due to the capacity to produce foaming water by the tree leaves and barks [61]. Saponins are secondary metabolites classified into triterpenoid and steroid glycosides, whose structures vary based on the number and position of attached sugar units [62]. These natural glucosides are amphipathic molecules composed of two distinct regions, a hydrophobic aglycone core and a hydrophilic sugar chain. The sugar moiety may include various units such as glucose, glucuronic acid, xylose, rhamnose, or methylpentose, which are connected to the aglycone via ether or ester linkages [62,63] (Figure 1A). These highly complex metabolites are present in a wide range of plant species and can be found in various plant parts including bark, leaves, stems, roots, and flowers [62,64,65]. This combination of polar and non-polar structural elements in their molecules explains their soap-like behavior in aqueous solutions [66,67] (Figure 1B).

Saponins are highly important in pharmacy, cosmetic, and food industries. Saponin metabolites are first isolated from soapwort (*Saponaria officinalis* L.) and have been traditionally employed as natural detergents in domestic applications [64,68]. In addition to their cleansing properties, they exhibit a wide range of biological activities, including immunoadjuvant, anti-inflammatory, antifungal, antiviral, molluscicide, hypoglycemic, hypocholesterolemia, and hemolytic effects [62,64,66,67]. The toxicity of certain saponins, closely associated with their hemolytic activity, is believed to stem from the aglycone’s high affinity for membrane cholesterol [64,66,69,70,71]. This interaction disrupts red blood cell membranes by forming pores through cholesterol complexation, leading to cell lysis [70,71,72,73].

### 2.2. Saponins as Immunoadjuvants

The adjuvant properties of these secondary metabolites were first noted in 1925, when it was shown that the addition of bread crumbs, tapioca, saponin, and “starch oil” to antigenic preparations greatly enhanced antibody responses to diphtheria or tetanus [11]. In 1951, Espinet used crude saponins to boost foot-and-mouth disease virus (FMDV) vaccine efficacy [74]. A major breakthrough came in 1974, when Dalsgaard successfully isolated Quil-A^®^ from the barks of the South American tree *Q. saponaria* Molina, demonstrating its ability to stimulate both humoral and cellular immune responses and induce differential antibody isotypes [36,67,75,76,77,78]. Since then, Quil-A^®^ has gained widespread use in veterinary vaccines and pre-clinical studies [67,79,80,81,82]. Additional studies showed its effects when co-formulated with aluminum salts, liposomes, and oil-in-water emulsions, with amphipathic proteins and lipids forming detergent/lipid/saponin complexes termed immune-stimulating complexes (ISCOMs) [83,84,85,86,87]. However, Quil-A^®^ remains a heterogeneous mixture—containing up to 23 saponin fractions detectable by reverse-phase high performance liquid chromatography (RP-HPLC)—and its toxicity limits its use in human vaccines [34,67,77,88].

**Figure 2:**
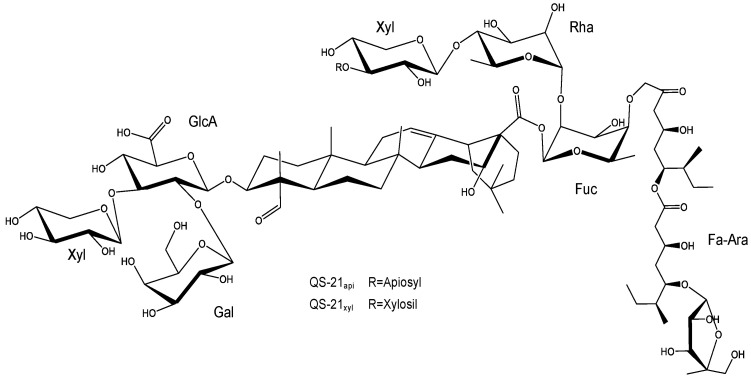
The QS-21 chemical structure. QS-21 is a complex triterpenoid saponin isolated from *Quillaja saponaria* bark that is characterized by four distinct structural domains. The molecule features a central lipophilic triterpene core (quillaic acid), which is flanked by a branched trisaccharide attached at the C3 position and a linear tetrasaccharide linked to the C28. Additionally, QS-21 contains a glycosylated pseudodimeric acyl chain (Fa-Ara) esterified to the fucose residue, contributing to its amphiphilic properties and adjuvant activity. QS-21 is as a mixture of two isomeric forms, QS-21-Api (65%) and QS-21-Xyl (35%), which differ only in the terminal sugar of the tetrasaccharide (apiose or xylose, respectively).

Afterwards, Kensil et al. purified several saponin fractions from Quil-A^®^ using RP-HPLC, identifying QS-7, QS-17, QS-18, and QS-21 (Figure 2), all of them with potent adjuvant activity (Figure 2) [77]. Among these, toxicity varied significantly. QS-18 was highly toxic in mice, whereas QS-7 and QS-21 were much less so [34,77,89]. Saponins from *Q. saponaria* bark, especially QS-21, promote Th1-type cytokines (IL-2, IFN-γ), stimulate CTL against exogenous antigens, and enhance IgG1, IgG2b, and IgG2a antibody production. These properties have attracted attention for their use as adjuvants in vaccines targeting intracellular pathogens and in therapeutic cancer vaccines [14,36,37,67,90,91,92,93,94,95].

**Figure 3:**
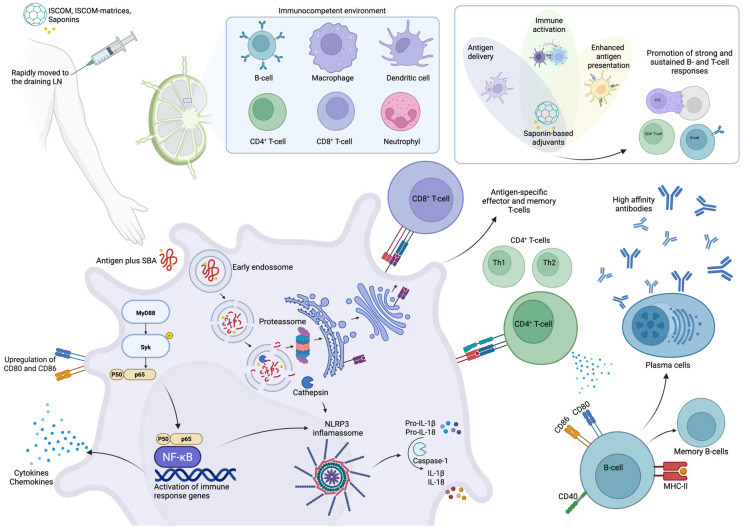
Schematic representation of the mechanisms underlying the adjuvant activity of Quillajaceae saponins. SBA induces the transient recruitment and activation of innate immune cells, facilitating antigen uptake and transport to draining lymph nodes. There, enhanced antigen presentation promotes the activation of CD4^+^ and CD8^+^ T cells. CD4^+^ T cells differentiate into Th1 and Th2 subsets and support B cell maturation, the development of long-lived antibody responses with high affinity. Simultaneously, SBA promotes the generation of memory T and B cells. After uptake by antigen-presenting cells, SBA components and antigens localize to lysosomes, where saponins disrupt the lysosomal membrane, enabling cytosolic translocation of the antigen. This supports the following dual antigen presentation: via MHC class II to CD4^+^ T cells and via cross-presentation on MHC class I to CD8^+^ T cells. SBA also induces co-stimulatory molecule expression (e.g., CD80/CD86) and activates the NLRP3 inflammasome, leading to the release of IL-1β and IL-18, along with other pro-inflammatory cytokines [96,97,99,112,115,116,117]. Created with BioRender.com.

The exact mechanism by which saponins in general act as adjuvants remains unclear, though recent studies have begun to clarify their modes of action [96,97,98,99]. Saponins can induce both Th1/Th2 immune responses (Figure 3). Structure–activity relationship studies (SAR) suggest that imine-forming carbonyl groups are essential for T cell activation and the subsequent induction of Th1/Th2 responses [67,100,101,102,103].

While saponins with diverse triterpenoid aglycones and oligosaccharide chains can activate DC, the presence of fucopyranosyl residues may skew the response toward a Th2 profile via interaction with the DC-SIGN receptor [103,104]. Glycosides like QS-21 (Figure 2) interact with both T cells and DC; the aldehyde group co-stimulates T cells, while the triterpene and fucosyl components engage DC [104,105,106]. The mechanism of QS-21 involves inducing CD8^+^ [67,107,108,109], partly due to a unique aldehyde group at the triterpene C4 position, believed to stimulate T lymphocytes and enhance Th1 responses [34,101,103,110,111,112]. Its lipophilic moiety further facilitates antigen delivery to APC, promoting endogenous processing and amplifying CTL production [48,103,111,113].

According to Lacaille-Dubois, QS-21 stimulates both Th2 (humoral) and Th1 (cell-mediated) immune responses through actions on antigen-presenting cells and T cells. It also activates the NLRP3 inflammasome, promoting the release of caspase-1-dependent cytokines IL-1β and IL-18 [99,108,112,114]. Ongoing research continues to reveal the complex signaling pathways involved in the immunomodulatory effects of saponins.

QS-21 is currently used in combination with other immunostimulants in adjuvant systems such as AS01 and AS02 [12,29,30,32,33,34]. AS01, designed to enhance CD8^+^ T cell responses, is used in two licensed human vaccines, Shingrix™ (herpes zoster) and Mosquirix™ (malaria), and in candidate vaccines for HIV and tuberculosis [29,32,108,118,119,120]. AS02, meanwhile, has been evaluated in vaccines targeting pathogens that require strong T cell and humoral responses, including hepatitis B, malaria, and HIV [121,122].

Despite its promise, QS-21 faces several limitations, including low yield, complex purification, limited natural availability, dose-limiting toxicity, and chemical instability [34,68,123,124,125]. To address this, chemical synthesis has enabled the production of pure, homogeneous QS-21 analogues with improved pharmacological profiles [125,126]. Initial semi-synthetic QS-21 variants were designed with stable amide linkages in the acyl chain domain, maintaining in vivo adjuvanticity while reducing toxicity [125,126].

Another strategy to facing these limitations involves mixing saponins with sterols, like cholesterol, to form micellar aggregates. Cholesterol is included in liposomes to bind and “detoxify” saponins, preventing pore formation in lipid bilayers, such as in erythrocytes [26,29,32]. More recently, Nuvaxovid™ (NVX-CoV2373), a recombinant spike protein COVID-19 vaccine, was approved for human use, and it employs Matrix-M^TM^, an ISCOM-like adjuvant composed of *Q. saponaria* saponin fractions [8,97,127]. Table 1 summarizes adjuvants derived from *Q. saponaria* that have been approved for human use, highlighting their formulations and applications in licensed vaccines.

**Table 1:** *Q. saponaria*-based adjuvants approved for human use.

	AS01	AS02	Matrix-M^TM^
**Composition and delivery system**	QS-21 + MPL in liposomes [27]	QS-21 + MPL in oil-in-water emulsion [27]	Saponin fraction from *Q. saponaria* in ISCOM-like nanoparticles [97]
**Immune response**	Th1 dominant response with CD8^+^ T cell activation and Ab responses [97]	Balanced T cell (Th1/Th2) and Ab responses [27]	Th1 dominant response with CD8^+^ T cell activation and Ab responses [97,118]
**Clinical applications**	Shingrix™ (Herpes Zoster); Mosquirix™ (Malaria); HIV, TB (candidate vaccines) [128]	Malaria, HIV, Hepatitis B (clinical trials) [128]	Nuvaxovid™ (NVX-CoV2373, COVID-19) [129]; Malaria [130]
**Advantages**	Efficient CD8^+^ activation; reduced QS-21 toxicity via liposomes	Broad immune stimulation; suitable for complex pathogens	Efficient CD8^+^ activation; self-assembling; reduced saponin toxicity via ISCOM-matrices
**Limitations**	Limited QS-21 supply; requires liposome formulation	Limited QS-21 supply; emulsion stability; potential reactogenicity	Limited saponin supply; formulation complexity

### 2.3. Quillajaceae Saponin-Based Nanoparticulated Adjuvants

Nanoparticle (NP)-based formulations have been used in medicine for nearly three decades, offering the passive targeting of APC by mimicking pathogens in size and shape, thereby enhancing antigen uptake, processing, and cross-presentation [117,131,132,133,134]. Various NPs (typically 20–200 nm in size) serve as vaccine antigen carriers, protecting antigens from degradation and promoting APC uptake. Common NP systems include VLP, self-assembled proteins, micelles, liposomes, ISCOMs, inorganic NP, and polymers [133,134,135,136,137].

ISCOMs are 40 nm cage-like complexes of Quil-A^®^, antigen, cholesterol, and phospholipids, designed to reduce saponin toxicity while preserving immunogenicity [138]. ISCOMATRIX™ (IMX) is a similar adjuvant, but it is assembled without antigen [83,84,139]. The goal of this saponin-based colloidal adjuvant formulation is to reduce the hemolytic effect inherent in saponins, retaining an effective antigen delivery system and adjuvant effect. Both are under clinical evaluation for influenza and HPV vaccines due to their ability to elicit strong antibody and T cell responses with reduced toxicity compared to pure QS-21 [87,97,116,130,140,141].

ISCOMs and IMX have been evaluated across multiple species, including small and large animals [82,83,96,142], and more recently in human clinical trials [116,130,140,143,144,145,146,147,148,149]. IMX can be performed or combined with antigens during formulation, offering flexibility in vaccine design. A key advantage of ISCOMs is their stability, retaining integrity for over a year at 4 °C. Although their exact mechanism remains unclear [150], clinical studies have shown ISCOM-based vaccines to be both safe and highly immunogenic [87,97,130,139,140,148,151,152,153]. Multiple preclinical and clinical studies confirm that ISCOMs and IMX elicit strong humoral, cellular, and innate immune responses across various antigens [97,142]. These adjuvants offer dual functionality, efficient antigen delivery and immune stimulation, resulting in robust, long-lasting effector and memory antibody and cellular responses [145,151].

Notably, unlike many adjuvants, IMX induces a mixed Th1/Th2 cytokine profile, supporting diverse antibody isotypes [154]. It rapidly, though transiently, stimulates serum levels of interferon-γ (IFN-γ) and interleukin-5 (IL-5), leading to strong antibody and CD8^+^ T cell responses [118]. High-frequency antigen-specific CD8^+^ T cell induction is a consistent hallmark of IMX-adjuvanted vaccines in both animal and human studies [97,102,112,116,149,155,156,157].

In 2011, Duewell et al. showed that Ovalbumin (OVA) adjuvanted with IMX triggers early inflammasome activation and a mixed Th1/Th2 response [156]. They also confirmed that IL-1β secretion in vitro is caspase-1-dependent. However, Wilson et al. reported distinct in vitro and in vivo mechanisms of IMX-induced inflammasome activation [98]. In vitro, IMX promoted IL-1β production in LPS-primed, thioglycolate-elicited peritoneal macrophages via NLRP3, ASC, and caspase-1/11, implicating lysosomal destabilization [98]. In contrast, in vivo NK cell activation by IMX depended on IL-18R but was independent of NLRP3 and IL-1R1. Adaptive immunity also relied on the IL-18 pathway, as IL-18^−^/^−^ and IL-18R^−^/^−^ mice showed reduced CD8^+^ T cell and IgG2c responses, whereas NLRP3 and IL-1R1 deficiencies had no such effect. TNF-α may serve as a natural priming signal compensating for the absence of a TLR agonist in IMX [98]. These findings highlight both inflammasome-dependent and -independent pathways of IMX-induced immunity and suggest that IL-18, possibly released through APC cell death at the site of injection or processed by alternative proteases like caspase-8, contributes to adaptive responses independently of NLRP3 and caspase-1 [114,158].

## 3. Saponins from *Q. brasiliensis* Leaves

### 3.1. Methods of Isolation, Structural Similarity, and Toxicity Concerns Among Q. saponaria Bark Saponins and Q. brasiliensis Leaves Saponins

**Figure 4:**
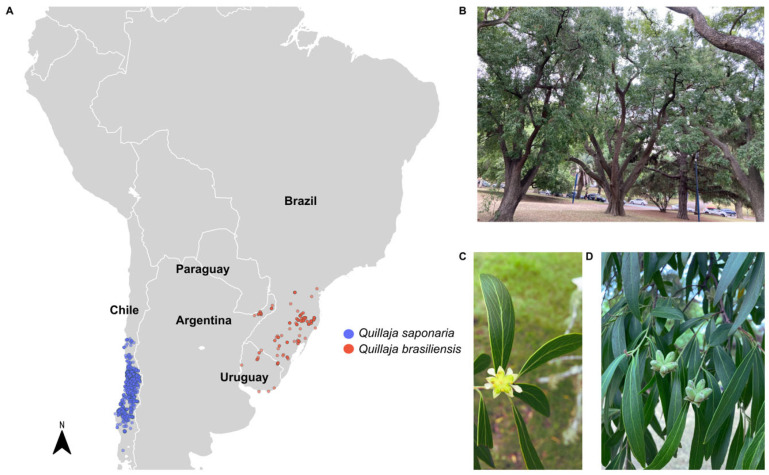
Geographic distribution of *Quillajaceae* and morphological aspects of *Q. brasiliensis*. (**A**) Occurrence records of *Q. saponaria* (blue) and *Q. brasiliensis* (red) in South America. Distribution data were retrieved from the Global Biodiversity Information Facility (GBIF, https://www.gbif.org, accessed on 1 July 2025), and the map was generated using Kauffman et al. 2004 [173]. Country borders are shown in white over a light gray political map. (**B**) Adult trees of *Q. brasiliensis* located in Parque Batlle, Montevideo, Uruguay. (**C**) Flower of *Q. brasiliensis*. (**D**) Fruits and leaves of *Q. brasiliensis* (Photos: F. Silveira).

The genus *Quillaja* exhibits a striking geographic separation in South America due to the Andes Mountains chain. *Q. saponaria* is native to Chile, while *Q. brasiliensis* thrives in the Araucaria forests of southern Brazil, Uruguay, northeastern Argentina, and eastern Paraguay [159,160] (Figure 4A). Due to the overharvesting of *Q. saponaria* barks in Chilean forests, which has led to significant ecological damage and supply [161,162], extensive efforts have been made in recent decades to discover new saponins with enhanced adjuvant activity and lower toxicity [67,163]. A promising alternative is *Q. brasiliensis*, now recognized as *Q. lancifolia*, a native species of southern Brazil known as “pau-sabão” (soap tree) due to the capacity of its leaves and bark to produce abundant foam in water [159,160,164]. It is considered a great tool of renewable alternative resource saponins, decreasing the pressure on *Q. saponaria* exploitation [162,165,165,166,167]. Figure 4B–D illustrates the trees, flowers, fruits, and leaves of *Q. brasiliensis*.

One method for obtaining saponins from *Q. brasiliensis* leaves, bark, and branches involves aqueous extraction followed by lyophilization. The resulting leaf extract can be further purified using reversed-phase silica gel (RP-C18) chromatography with a methanol gradient. A notable saponin fraction obtained via this method, eluted at 90% methanol, is known as QB-90 [166,168,169,170]. Recently, a detailed method for purifying saponins from *Q. brasiliensis* leaves using RP-C18 chromatography was reported, describing the preparation of aqueous extracts and the isolation of QB-90 [171].

Building on these advances, several studies have isolated and studied the raw aqueous extract (AE) and RP-C18 fractions, including the saponin-enriched fraction QB-90, QB-80, Fraction B, and Fraction 3 [166,172]. The QB-80 fraction, a saponin-enriched fraction, shares structural similarities with QB-90 and was obtained using the same purification protocol [14], but eluted at 90% of methanol. Subsequently, two alternative methods were developed for isolating bioactive saponins. Fraction B was obtained from the AE of air-dried leaves via solid-phase extraction on C-18 reversed-phase columns, using a stepwise methanol–water gradient. Fraction 3 was isolated from leaf AE through a combination of ultrafiltration and ion-exchange chromatography [172].

Initial studies on *Q. brasiliensis* leaf constituents were conducted by Kauffman et al. (2004) [173], who reported the isolation of a new abietane diterpene (9-O-beta-D-glucopyranoside of 16-hydroxylambertic acid), a prosapogenin (3-O-beta-D-glucuronopyranosyl-quillaic acid), and two flavonoids (quercetin and rutin). They also described the preparation of aqueous extracts from leaves, bark, and branches and isolated the QB-90 fraction from leaves, characterizing its structure through hydrolysis and NMR analyses [168,173]. ^1^H NMR spectra of QB-90 and aqueous extracts from leaves, barks, and branches closely resembled those of Quil-A^®^, highlighting notable structural similarities between the saponins of *Q. brasiliensis* and *Q. saponaria* [168]. Further studies by Cibulski et al. using MALDI-ToF–MS (matrix-assisted laser desorption/ionization time-of-flight mass spectrometry) [14,174] and DI-ESI-MS [175] confirmed these similarities in saponin composition between the two species. More recently, Wallace et al. identified 75 distinct saponins in *Q. brasiliensis.* From these, 21 were from leaves, and 54 were from bark [176]. Among these, a novel triterpene saponin, Qb1 (an unreported isomer of QS-21), was identified and elucidated by MS and NMR techniques [174,176].

**Figure 5:**
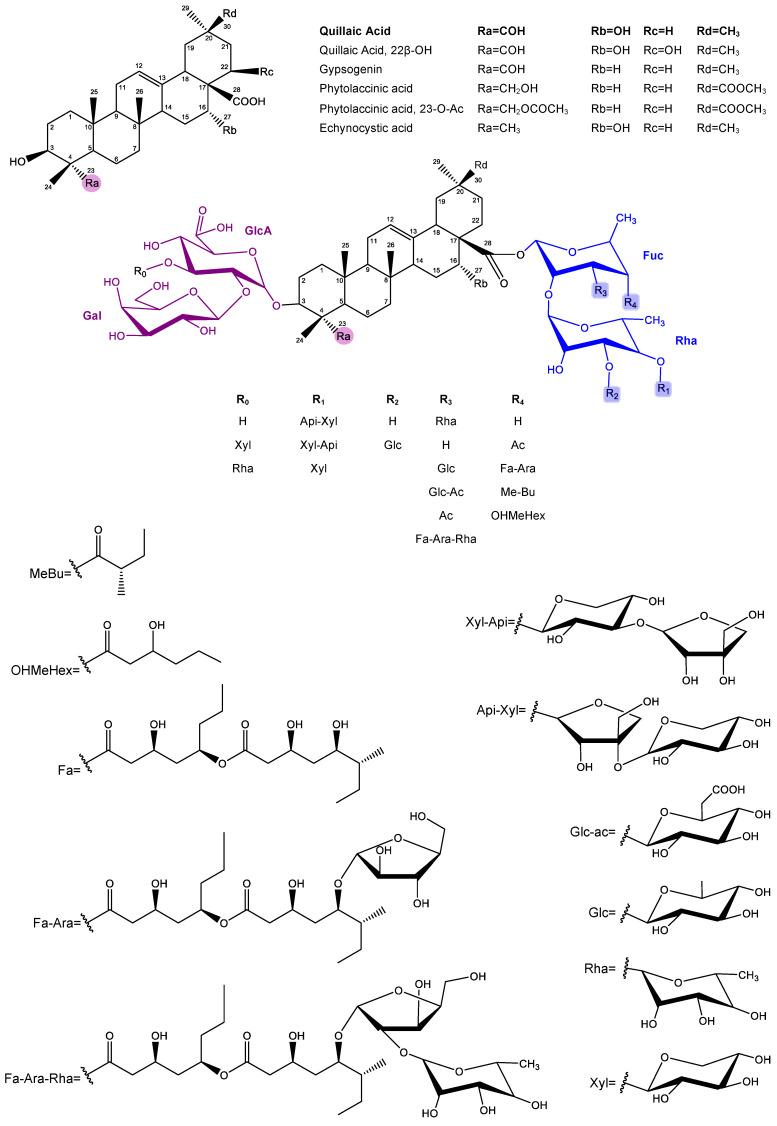
General structure of saponins from *Q. saponaria* and *Q. brasiliensis***.** Saponins of *Quillajaceae* contain a triterpenic aglycone, most frequently quillaic acid, and are glycosylated at the C-3 and C-28 positions of the aglycone.

The saponins isolated from the leaves and bark of *Q. brasiliensis* present complex triterpene structures, which demonstrate remarkable similarity with the classical saponins of *Q. saponaria*, especially regarding the nature of the aglycones and the organization of the sugar residues. Both species share bidesmosidic saponins, with oligosaccharides linked at the C-3 and C-28 positions of the aglycone, usually quillaic acid, phytolaccinic acid, or their derivatives. In particular, the substitution pattern observed in the sugar residues, such as rhamnose, fucose, xylose, glucuronic acid, and acylations with groups such as Fa-Ara, reinforces the structural similarity between the saponins of both species. Mass spectrometry analysis (LC-ESI-IT-MSⁿ) showed that the fragment ions (“A ions”) of the saponins from *Q. brasiliensis* present the same fragmentation profiles already described for *Q. saponaria*, indicating the conservation of the basic architecture of the compounds. Additionally, many of the compounds identified in *Q. brasiliensis* have molecular masses identical or very close to those of the known saponins from *Q. saponaria* and are considered homologous or have variants with specific modifications such as substitutions of sugars or acyl groups. These structural similarities explain the functional equivalence observed between the extracts of the two species, especially in the context of their immunoadjuvant activity. Notably, the QS-21 *m*/*z* ion was detected in nanoparticles formulated with raw aqueous extracts (AE) [174] as well as with QB-80 [175] and Fraction B [177], all derived from *Q. brasiliensis* leaves. In mass spectrometry, *m*/*z* denotes the mass-to-charge ratio of an ion, defined as the ion’s mass (*m*) divided by its net charge (*z*), and serves as the principal variable by which ions are separated and detected within the mass analyzer. The general structures of the *Quillajaceae* saponins are described in Figure 5.

The primary limitation of saponins as adjuvants is their potential toxicity of amphiphilic molecules due to their ability to interact with cholesterol in mammalian cell membranes, disrupting membrane integrity and function [77,178], which is commonly assessed through a range of in vitro and in vivo assays [178]. A standard initial evaluation involves measuring hemolytic activity against red blood cell suspensions. Silveira et al. (2011) reported that saponins extracted from *Q. brasiliensis* plants in Uruguay (designated as QB-90U) exhibited lower hemolytic activity than Quil-A^®^ [14,91]. Further cytotoxicity assessments were conducted on Vero cells using MTT viability and lactate dehydrogenase (LDH) release assays [86,91], aligning well with previous in vivo toxicity data in mice [14,86,168]. Additionally, Cibulski et al. (2016) demonstrated that both QB-90 and aqueous extracts (AE) from *Q. brasiliensis* exhibited significantly lower hemolytic activity (higher HD_50_ values) than Quil-A^®^ [14,90].

Injection site reactions are the most frequently reported manifestation of vaccine-associated toxicity in animal models. These local responses, common with parenteral vaccines, can range from pain and swelling to granulomas, sterile abscesses, and ulceration [179]. Consistent with this, the in vivo toxicity of *Q. brasiliensis* saponins has been evaluated in several studies. Fleck et al. (2006) conducted the first acute toxicity assay using the raw AE and QB-90 in CF-1 female Swiss mice via subcutaneous (s.c.) administration [168]. No lethality, swelling, or hair loss effects were observed [168]. However, bark extracts caused local swelling at doses above 400 µg/dose following a second administration.

Cibulski et al. (2016) [86] further assessed acute toxicity in CD-1 male mice. At the lowest tested dose (31.25 µg), QB-90 induced no signs of local toxicity or lethality, while Quil-A^®^ caused 40% lethality. At the highest dose (125 µg), mortality reached 60% with QB-90 and 100% with Quil-A^®^, reinforcing that QB-90 is significantly less toxic than Quil-A^®^, both in vitro and in vivo.

In summary, semi-purified aqueous extracts and saponin-enriched fractions from *Q. brasiliensis* demonstrate lower toxicity compared to Quil-A^®^, despite their structural similarity. These differences may be attributed to subtle structural variations and differences in extraction and purification methods [91].

### 3.2. Q. Brasiliensis Saponins: A Potent Natural Immunoadjuvant

A descriptive analysis of the immune responses elicited by diverse SBA derived from *Q. brasiliensis* was conducted using data compiled from studies involving different viral antigens and administration routes. These data are shown in Table 2. The adjuvants assessed include AE, QB-90, and QB-80 and its nanoparticulate formulations, as well as other purified saponin fractions. These were evaluated primarily through subcutaneous and intranasal immunization routes in murine models, using diverse inactivated or recombinant antigens such as herpesvirus (BoHV1 and BoHV5), poliovirus, pestivirus (BVDV), rabies virus, Zika virus, and influenza virus A.

**Table 2:** Immunoadjuvant activity of *Q. brasiliensis* saponins.

Adjuvant/Route (μg/Dose)	Antigen	IgG	IgG1	IgG2a/2c	IgG2b	IgG3	IgA	NAb/HAI	DTH	Other Assays	Ref.
AE-sc (400)	Herpesvirus (BoHV1, inactivated virus)	*	*	*							[168]
QB-90-sc (200, 150, 100 and 50)		*	*	*							
QB-90 ^a^-sc (100)	Herpesvirus (BoHV5, inactivated virus)	**	*	***	***	**			***	Upregulation of IFN-γ and IL-2 mRNA in splenocytes	[91]
AE-sc (400)	Poliovirus (Sabin 1, inactivated virus)	**	**	**			*		**	Upregulation of IFN-γ and IL-2 mRNA in splenocytes	[92]
QB-90-sc (50)		**	**	**			*		**		
AE-sc (400, 200 and 100)	Pestivirus (BVDV, inactivated virus)	***	***	***					***	Splenocyte proliferation (including CD8^+^ T cells) and IFN-γ, IL-2, TNF, IL-10, and IL-17 cytokines secretion	[14,90]
QB-90-sc (100, 50 and 10)		***	***	***					***		
QB-90-sc (10)	Ovalbumin (OVA)	***	**	-					-	Splenocyte proliferation; IFN-γ, IL-2 secretion in IQB-90 vaccines	[86]
IQB-90-sc (10)		***	***	**					***		
QB-90-in (2)		-	-	-			-		-		
IQB-90-in (2)		***	***	-			*/*** ^b^		-		
AE-sc (400)	Rabies virus (inactivated virus)	*	*	*					*	Protection in a challenge assay provided by the adjuvanted anti-rabies vaccines	[172]
QB-80-sc (100 and 50)		*	*	*					*		
QB-90-sc (100 and 50)		*	*	*					*		
Fraction B-sc (50)		*	*	*							
Fraction 3-sc (50)		*	*	*							
AE-sc (400 and 200)	Pestivirus (BVDV, inactivated virus)	****	****	***					****	Antigen-specific IFN-γ production in CD4^+^ and CD8^+^ T cells; dose sparing effect	[14]
QB-80-sc (100, 50 and 10)		****	****	****					***		
IMXQB-80-sc (2.5)	Zika virus (inactivated virus)	***	****	***				***		No differences between IMXQB-80 (2.5 μg) and QB-80 (10 μg) immunized animals	[175]
QB-80-sc (10)		****	***	****				***			
IQB80-sc (10 and 2)	Zika virus (recombinant E protein)	****	**	**	****	***		***		Antigen-specific splenocyte proliferation and antibody avidity increase	[94]
IQB90-sc (5)	Influenza (seasonal split vaccine)	****	**	**	**	****	-	****	**	Protection in a challenge test provided by adjuvanted vaccines; dose sparing effect	[93]
IQB90-in (5)		****	****	****	****	****	**** ^b^	****			
IMXQB-sc (5)	Influenza (seasonal split vaccine)	****	***	****	***	***		*		Protection in a challenge test provided by adjuvanted vaccines; high levels of NAb and improved serum HI	[180]
IMXQB-in (2.5)		***	***	***	***	***	***	***			
IMXQB-old mice sc (5)	Influenza (seasonal split vaccine)	**	*	-				-		Protection in a challenge test provided by adjuvanted vaccines; IgG1, IgG2a, and IgA are maintained 120 days after priming	[181]
IMXQB-old mice in (2.5)		***	**	*			****	***			

IQB: vaccines as ISCOM, IMXQB: vaccines adjuvanted with ISCOM-matrices, sc subcutaneous, in intranasal, ^a^ QB-90 derived from Uruguayan specimens. ^b^ Seric IgA. Nab/HAI (neutralizing antibodies) DTH (delayed-type hypersensitivity), - not significant, * *p* < 0.05, ** *p* < 0.01, *** *p* < 0.001, **** *p* < 0.0001 (against group of animals vaccinated with unadjuvanted formulation).

The results across studies emphasize the versatility and efficacy of SBA derived from leaves of *Q. brasiliensis* in eliciting balanced and protective immune responses across different viral vaccine candidates. Their effectiveness via mucosal routes and in aged models further supports their potential for application in both human and veterinary vaccine development

#### 3.2.1. Humoral Response

Fleck et al. (2006) [168] were the first to assess the adjuvant potential of *Q. brasiliensis* saponin-enriched fractions, which closely resemble those from *Q. saponaria*. A purified leaf-derived saponin fraction (QB-90) effectively stimulated both Th1 and Th2 responses, as well as cytotoxic T cell activity against herpesviruses 1 and 5 in mice, with a response comparable to *Q. saponaria* saponins (Quil-A^®^) [91,168]. Similarly, QB-90 from Uruguayan plants (QB-90U) induced strong IgG responses with neutralizing antibodies against BoHV-5 [91]. The immunoglobulin isotype profile elicited by the Uruguayan QB-90U fraction was comparable to that induced by Quil-A^®^, with a Th1-biased response characterized by elevated levels of IgG2a and IgG3, alongside significant IgG1 production, indicating concurrent Th2 activation [182]. AE from bark, leaves, and branches also showed adjuvant activity on par with Quil-A^®^ in experimental BoHV-1 vaccines [168]. Additionally, AE from *Q. brasiliensis* leaves has been tested with human poliovirus, bovine viral diarrhea virus (BVDV), and inactivated rabies virus in mice [14,90,92,168,172].

Recent investigations have demonstrated that novel saponin fractions from *Q. brasiliensis*, such as QB-80 and Fraction 3, exhibit potent immunostimulant properties when co-administered with inactivated rabies virus antigens, eliciting robust antibody responses in murine models [172]. Cibulski et al. (2016) further reported that both AE and QB-90 fractions significantly enhanced the humoral response against BVDV, primarily by increasing total IgG levels via increments of IgG1 (Th2-associated) and IgG2a (Th1-associated) isotypes [90].

Chemically, both QB-80 and AE display notable similarities to the commercial saponin Quil-A^®^, though with reduced toxicity. These fractions not only induced sustained IgG1 and IgG2a responses with high avidity but also promoted delayed-type hypersensitivity (DTH) reactions and increased IFN-γ secretion in both CD4^+^ and CD8^+^ T cell populations [14]. As well as due to its low cost and ease production, AE emerges as a promising candidate for industrial-scale applications, including nanoparticle-based vaccine formulations [174].

In the context of Zika virus immunizations, both QB-80 and its nanoparticle-based formulation (IMXQB-80) significantly elevated anti-Zika virus IgG subtypes (IgG1, IgG2b, IgG2c) and neutralizing antibody titers, outperforming unadjuvanted vaccines [175]. Notably, IMXQB-80 achieved comparable immunogenicity with fourfold less saponin content and reduced toxicity. Mice immunized with the IQB80-zEDIII formulation exhibited enhanced IgG subclass responses, strong neutralizing activity, and robust splenocyte proliferation, supporting its candidacy for Zika virus vaccine development [94].

In studies on influenza, an ISCOM-like nanoparticle formulation incorporating QB-90 (IQB90) demonstrated superior protective efficacy compared to unadjuvanted vaccines, whether delivered subcutaneously or intranasally. The IQB90 formulation not only induced higher hemagglutination inhibition titers but also exhibited a tenfold dose-sparing effect, an attribute of significant relevance for pandemic preparedness strategies [93,180].

#### 3.2.2. Cell-Mediated Immune Response Induced by *Q. brasiliensis* Saponins

DTH is a classical in vivo indicator of cell-mediated immunity, marked by the recruitment and activation of macrophages and T lymphocytes, particularly CD4^+^ and CD8^+^ cells, along with the release of pro-inflammatory cytokines such as IL-1, IL-2, IFN-γ, and TNF-α [183,184,185,186]. In mice, DTH reactions typically emerge 16–24 h post-antigen challenge, manifesting as mononuclear and granulocytic infiltration, increased vascular permeability, and edema [184].

Experimental vaccines adjuvanted with *Q. brasiliensis* saponin fractions (QB-90, QB-80, and the raw AE) induced significantly stronger DTH responses compared to control formulations (Alum or Quil-A^®^), particularly in response to bovine herpesvirus and human poliovirus antigens [91,92]. These results suggest the activation of memory Th1 CD4^+^ T cells [184], consistent with the immunostimulatory profile of Quil-A^®^ [187].

In further studies, vaccines formulated with QB-90, QB-80, or AE, in combination with rabies (Pasteur strain) or BVDV antigens, also produced pronounced DTH responses relative to unadjuvanted controls [90,172]. Notably, the IQB90-Flu nanoparticle vaccine elicited robust DTH reactions at 24 h in both wild-type and caspase-1/11 knockout mice, while no DTH responses were observed in animals receiving the unadjuvanted influenza vaccine [93].

The cytokine profile induced by QB-90U paralleled that of Quil-A^®^, favoring a Th1-biased response characterized by elevated IgG2a and IgG3 isotypes, as well as increased IFN-γ and IL-2 mRNA expression in splenocytes [90,91,92]. Additional analysis using a Th1/Th2/Th17 bead array showed that BVDV-stimulated splenocytes from mice immunized with QB-90-adjuvanted vaccines secreted high levels of IFN-γ, TNF-α, and IL-2, corroborating the Th1-polarized humoral response. QB-90 also promoted IL-10 and moderate levels of IL-4 and IL-6, indicating a mixed Th1/Th2 cytokine milieu [90]. De Costa et al. confirmed these findings through qPCR, demonstrating nearly tenfold upregulation of IFN-γ transcripts in splenocytes from mice immunized with QB-90 or AE compared to non-adjuvanted controls, matching levels induced by Quil-A^®^ [92].

Cell-mediated immunity, particularly through CD8^+^ T cells, plays a critical role in viral clearance by recognizing viral peptides presented via MHC I and initiating cytolytic activity or cytokine-mediated inhibition [188,189,190,191]. Mice immunized with BVDV antigens adjuvanted with AE or QB-90 exhibited significantly enhanced BVDV-specific CD8^+^ T cell proliferation and increased frequencies of IFN-γ^+^ CD4^+^ and CD8^+^ T cells, as assessed by flow cytometry [90]. These findings suggest that QB-90 enhances antigen cross-presentation, potentially via the chemotactic recruitment of dendritic cells to the immunization site [90].

#### 3.2.3. Mucosal Immunity Induced by *Q. brasiliensis* Saponins

Mucosal surfaces, lining the gastrointestinal, respiratory, and urogenital tracts, represent major entry points for pathogens. Despite this, most vaccines are delivered parenterally, with few licensed for mucosal administration due to the absence of effective delivery systems and mucosal adjuvants [192,193,194]. Current adjuvants show limited efficacy in enhancing mucosal immune responses.

Intranasal vaccination promotes local immune responses, including secretory IgA and the induction of resident memory B and T cells, reducing infection rates and viral shedding [192,195]. Intranasal immunization with adjuvanted inactivated vaccines effectively induces both mucosal IgA, offering strong cross-protection against variant viruses in the upper respiratory tract, and serum IgG, providing protection in the lower tract [196]. Compared to injectable vaccines, nasal vaccines elicit higher local IgA levels and stronger mucosal cell-mediated immunity, albeit with lower systemic antibody titers. Nevertheless, both routes demonstrate comparable efficacy (70–90%) in healthy individuals when the vaccine and epidemic virus are well-matched antigenically [197].

Studies using *Q. brasiliensis*-derived saponins, such as QB-90, demonstrated enhanced mucosal immunity. De Costa et al. showed that intranasal immunization with the QB-90-adjuvanted poliovirus antigen induced high IgA titers in bile, feces, and vaginal washings, comparable or superior to Quil-A^®^ [92]. Additionally, particulate formulations like IQB-90 promoted both mucosal IgA and systemic IgG responses across multiple sites, including the nasal passages, large intestine, and vaginal lumen [86].

Recent findings revealed that intranasal delivery of an inactivated seasonal influenza vaccine adjuvanted with IQB-90 enhanced IgM, IgG, and IgA levels 28 days post-priming. This experimental formulation also induced higher IgG1, IgG2a, IgG2b, and IgG3 levels and elevated hemagglutination inhibition titers compared to unadjuvanted commercial vaccines [93].

#### 3.2.4. ISCOMs and ISCOM-Matrices Based on *Q. brasiliensis* Saponins

**Figure 6:**
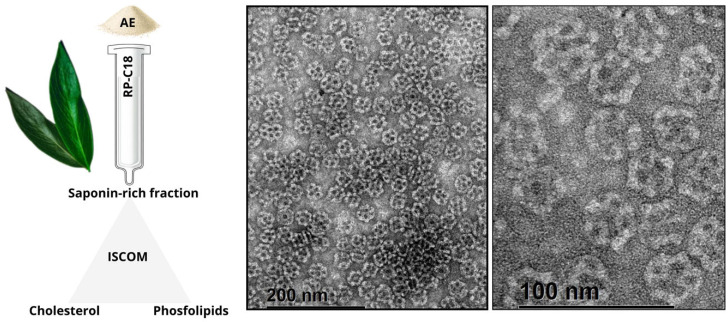
Preparation and ultrastructural visualization of *Q. brasiliensis*-derived ISCOM-matrices. On the **left** side, there is an illustrative scheme of the process involved in obtention of the raw aqueous extract (AE) of *Q. brasiliensis* leaves and the reverse-phase chromatography (RP-C18) used to obtain a saponin-rich fraction. The formulation of ISCOMs involves the combination of the saponin-rich fraction with cholesterol and phospholipids, resulting in nanostructures as visualized by transmission electron microscopy (TEM) in the images on the right. For these images, ISCOM-matrices were placed on formvar carbon grids (300 mesh) and negatively stained with 2% uranyl acetate and examined with a JEM-2100 TEM operated at an 200 kV accelerating voltage [198]. Scale bars: 200 nm (**middle**) and 100 nm (**right**).

An improvement to the use of saponins as adjuvants was introduced by the development of immune stimulating complexes. These have been used as antigen delivery systems that proved to exert powerful immune stimulating activities yet displaying reduced toxicity in several animal models. Prior to the study by Cibulski et al. [86], there were no reports of ISCOMs production using saponins extracted from any plant other than *Q. saponaria*. In the study, for the very first time, ISCOM formulations were constructed by replacing the Quil-A^®^ component by QB-90. These saponins extracted from *Q. brasiliensis*, successfully formulated ISCOMs, preserve their adjuvant activity while enhancing stability and reducing toxicity, notably by eliminating hemolytic effects [86,94,175]. Figure 6 provides a detailed illustrative scheme of the process for obtaining *Q. brasiliensis*-derived ISCOM-matrices, from the extraction of the raw aqueous extract (AE) to the final formulation and visualization by transmission electron microscopy (TEM).

The immunogenicity of ISCOMs prepared with the QB-90 fraction (IQB-90) was comparable to classical Quil-A^®^-based ISCOMs (IQA). IQB-90 nanoparticles (~40–50 nm, spherical, cage-like particles) comprise QB-90, cholesterol, phospholipids, and antigen (e.g., ovalbumin). These nanoparticles were efficiently internalized in vitro by murine bone marrow-derived DC and, upon subcutaneous administration, elicited strong serum IgG1 and IgG2a responses, DTH reactions, T cell proliferation, and Th1 cytokine production (IFN-γ, IL-2). Intranasally delivered IQB-90 also induced serum IgG/IgG1 and mucosal IgA responses across distant mucosal sites [86].

Mechanistic studies on ISCOM-matrices adjuvant based on QB-90 (IMXQB-90) revealed rapid cytosolic antigen delivery in dendritic cells, broad cytokine and chemokine induction, and the bridging of innate and adaptive immunity via a MyD88-dependent, Toll-like receptor-independent pathway [115,116,199]. Furthermore, QB-90 and IMXQB-90 promoted immune cell recruitment to draining lymph nodes and spleen, stimulated IL-1β secretion through caspase-1/11 and MyD88 pathways, implying canonical inflammasome activation, and induced the upregulation of cytokine and chemokine gene expression [99].

Cibulski et al. demonstrated that replacing Quil-A^®^ with QB-90 in ISCOM and ISCOM-matrices formulations (IQB-90 and IMXQB-90) maintained potent immunogenicity, including robust antibody production, myeloid and lymphoid cell activation, and Th1-type cytokine secretion (IFN-γ, TNF-α) [86,99]. In vitro, both formulations triggered caspase-1-dependent IL-1β production in bone marrow-derived dendritic cells. However, the role of inflammasome activation in vivo remains to be fully elucidated [158].

Recent studies employing Zika virus antigens confirmed the efficacy of QB-90-based adjuvants. Both a classic inactivated vaccine and a recombinant antigen formulation adjuvanted with IMXQB-80 or IQB80-zEDIII induced significantly higher anti-Zika virus IgG titers and neutralizing antibodies compared to unadjuvanted controls [94,175]. Additionally, a split-inactivated influenza vaccine adjuvanted with IQB-90 elicited stronger protective responses than a commercial unadjuvanted vaccine when administered via either subcutaneous or intranasal routes [93].

In line with these findings, Silveira et al. [180] demonstrated that a trivalent influenza vaccine (TIV) formulated with *Q. brasiliensis*-based IMXQB nanoparticles induced potent humoral and cellular immune responses when delivered subcutaneously or intranasally, including high levels of IgG1/IgG2a, Th1/Th2 cytokines, and effector T cells. Notably, intranasal immunization with TIV-IMXQB conferred full protection against a lethal viral challenge, preventing weight loss, lung viral replication, and mortality.

Further supporting these observations, a subsequent study in aged mice revealed that IMXQB-adjuvanted TIV significantly improved both systemic and mucosal immunity when administered subcutaneously or intranasally [181]. The adjuvanted vaccine elicited sustained IgM, IgG, and IgA responses, enhanced hemagglutination inhibition (HAI) titers, and accelerated recovery following viral challenge. These findings highlight the potential of *Q. brasiliensis*-derived IMXQB as a versatile and effective mucosal and systemic adjuvant platform, particularly relevant for vulnerable populations such as the elderly.

## 4. Conclusions

Saponins are powerful adjuvants that enhance cellular immunity, making them particularly valuable for vaccines against intracellular pathogens, viruses, and cancers [36,67,75,76,77,78]. While *Q. saponaria* bark extract semi- or purified fractions have been widely used in both veterinary and human vaccines [97,98,116,118], their overharvesting has caused ecological damage in Chilean forests [162,165]. This has driven the search for sustainable alternatives with comparable immunostimulatory properties but reduced toxicity [67,163].

*Q. brasiliensis*, native to South America, has emerged as an ecologically sustainable solution [166,167]. Its leaf-derived saponins share structural and functional similarities with *Q. saponaria* saponins while offering several advantages [168]. Fractions like QB-90, QB-80, and the aqueous extract demonstrate potent adjuvant activity, inducing balanced humoral (IgG1, IgG2a) and cellular (Th1/Th2/Th17, CTL) responses with improved safety profiles. When incorporated into nanoparticle platforms (ISCOMs/ISCOM-matrices), *Q. brasiliensis* saponins show enhanced efficacy against diverse pathogens, including influenza, Zika, and rabies, while minimizing hemolytic effects. Their mechanism of action involves NLRP3 inflammasome activation, dendritic cell recruitment, and cytokine production (IFN-γ, IL-1β) and can be particularly valuable for adjuvant vaccines to diseases requiring strong cellular immunity (e.g., malaria, tuberculosis, HIV).

From an economic standpoint, the sourcing and production of saponins present significant challenges and opportunities for vaccine development. Currently, the market price of Quil-A^®^ exceeds USD 500 per gram, while highly purified QS-21 can cost over USD 300 per milligram, making these adjuvants among the most expensive components of vaccine formulations. In contrast, *Q. brasiliensis* saponins can be sustainably harvested from leaves, avoiding bark extraction and thus minimizing ecological impact. Moreover, leaf collection allows for more frequent and scalable harvesting cycles. Although no commercial suppliers of *Q. brasiliensis* saponins currently exist and market pricing has yet to be established, preliminary studies indicate that its saponin fractions exhibit immunoadjuvant properties comparable to those of *Q. saponaria* [168]. As such, *Q. brasiliensis* represents a promising avenue for future development of accessible, sustainable, and economically viable vaccine adjuvants, particularly in settings where cost is a critical factor.

The sustainable leaf-harvesting approach for *Q. brasiliensis* addresses both ecological concerns and supply chain limitations, positioning it as a strategic resource for next-generation vaccine development. Ongoing preclinical and clinical studies will be crucial to fully realize its potential in human and veterinary applications, offering a promising combination of immunogenicity, safety, and environmental protection.
